# Managing the Health Impacts of Drought in Brazil

**DOI:** 10.3390/ijerph111010737

**Published:** 2014-10-16

**Authors:** Aderita Sena, Christovam Barcellos, Carlos Freitas, Carlos Corvalan

**Affiliations:** 1 Institute of Health Communication and Information (ICICT), Oswaldo Cruz Foundation (Fiocruz), Av. Brasil 4365, Rio de Janeiro, RJ 21045-900, Brazil; E-Mails: aderitasena@gmail.com (A.S.); xris@fiocruz.br (C.B.); 2 National School of Public Health (ENSP), Oswaldo Cruz Foundation (Fiocruz), Av. Brasil 4036, RJ 21040-361, Brazil; E-Mail: carlosmf@ensp.fiocruz.br; 3 Pan American Health Organization and World Health Organization (PAHO/WHO), Brasilia 70800-400, Brazil

**Keywords:** drought, climate change, decision-making, governance, adaptation, adaptive capacity, health determinants, vulnerability

## Abstract

Drought is often a hidden risk with the potential to become a silent public health disaster. It is difficult to define precisely when it starts or when it is over, and although it is a climatological event, its impacts depend on other human activities, and are intensified by social vulnerability. In Brazil, half of all natural disaster events are drought related, and they cause half of the impacts in number of affected persons. One large affected area is the semiarid region of Brazil’s Northeast, which has historically been affected by drought. Many health and well-being indicators in this region are worse than the rest of the country, based on an analysis of 5565 municipalities using available census data for 1991, 2000 and 2010, which allowed separating the 1133 municipalities affected by drought in order to compare them with the rest of the country. Although great progress has been made in reducing social and economic vulnerability, climate change and the expected changes in the semiarid region in the next few decades call for a review of current programs, particularly in public health, and the planning of new interventions with local communities. This study reviews the literature, analyzes available data and identifies possible actions and actors. The aim is to ensure there will be sufficient and sustainable local adaptive capacity and resilience, for a population already living within the limits of environmental vulnerability.

## 1. Drought, a Silent Public Health Disaster

Drought is largely a hidden risk and its health impacts are poorly recorded internationally. Every year, prolonged drought disasters affect several million persons. Between 1960 and 2013, 612 drought events resulted in 2.19 million deaths and 2.14 billion affected persons. Since 1990, 373 events recorded resulted in 4272 deaths and 1.17 billion affected persons [1]. The general trend is of increasing number of events and affected persons per year, but with a reduction in fatalities. However, drought has human health and well-being impacts that are hard to measure accurately. Drought can have impacts on known health risk factors such as inadequate or unsafe water for consumption and sanitation, increased population displacement, and disruption of local health services. It also impacts on acute and chronic health effects including malnutrition, increased risk of communicable diseases, respiratory conditions, psycho-social stress and mental health disorders [2,3,4,5,6,7].

Drought is a type of climatological process defined by spatial and temporal limits. It affects permanently large areas of the planet, characterized as semiarid or suffering from desertification, as well as humid areas during specific seasons or prolonged over years. As a risk and disaster, it is constructed by economic decisions and social choices. Meteorological drought (generally manifested as precipitation deficiency) is a climatic phenomenon, which becomes hazardous when it results in agricultural (soil moisture deficiency) or hydrological (surface and subsurface water deficiency) drought, depending on other social and economic determinants other than just rainfall [8]. Different from other climate related events, drought appears slowly and silently, without showing visible impacts in the short term. The precise time of onset or its end are not easily defined. This lack of visibility, awareness and characterization of the risks can lead to much human suffering and great economic losses at the local level, as in the case of small-scale farming or subsistence agriculture [9]. Although drought is defined as a climatological event, it is also worsened by human activities. Examples of these include population growth and movement, land use change, unsustainable economic growth, inadequate infrastructure and inadequate water resource management [10].

Another special factor of drought is that the impacts can last for years, and although it may cover several countries, these countries would feel the impacts at different degrees depending on the region and affected population, where the poor and vulnerable populations tend to suffer the greatest consequences [11,12]. Prolonged drought in a developing country could result in malnutrition, population displacement and loss of lives, while in a developed country it would result mostly in economic losses [13]. Less is known about drought impact on chronic non-communicable diseases and mental health, especially in developing countries. A recent review of the literature on climate change and mental health also touches on extreme events such as droughts. The authors propose a framework separating acute weather events (such as hurricanes) and sub-acute events where droughts are included. For the latter, direct mental health concerns include chronic stress, elevated rates of violence and aggression. Indirect effects on mental health may occur through a complex interaction of physical health impacts and damage to livelihoods, leading to elevated rates of chronic mood disorders and even suicide [14].

Gender differences in the management and impact of drought also need special attention. A study of drought in Brazil linked mental health with gender differences and observed higher levels of anxiety in women living in drought affected areas. This is likely the consequence of women’s drought related impaired role as producers and providers. Men appeared more emotionally distressed than counterparts in areas not affected by drought. Drought was seen as a driver for men to migrate to other areas in search for jobs, increasing both their own and their family’s stress and anxiety levels [15]. The United Nations Convention to Combat Desertification (UNCCD) has from its creation understood the differentiated roles of women and men in the management of natural resources, such as land and water [16]. At the 10th session of the Conference of the Parties to the UNCCD in 2011, an Advocacy Policy Framework on Gender was adopted, with the aim of addressing the drivers of land degradation and promoting gender equality. Specifically, the Policy promotes partnerships, capacity building, equal access to education and health care, and women’s right and ownership of land [17]. Therefore, understanding the complex issues surrounding drought, including the social, economic, environmental and health characteristics of the population are needed to ensure an effective process of disaster risk management.

UNCCD defines “desertification” as land degradation in arid, semiarid and dry sub-humid areas resulting from various factors, including climatic variations and human activities; and “drought” as the naturally occurring phenomenon that exists when precipitation has been significantly below normal recorded levels, causing serious hydrological imbalances that adversely affect land resource production systems [16]. These definitions also point to food security as the main issue, and therefore it neglects to address other key factors, besides agriculture, which contribute to ill health. In their guides for climate change vulnerability and adaptation assessments, the World Health Organization adopted a definition of “risk” as a product of the likelihood of expose (e.g., to an extreme climatic event) and the consequences of that exposure; and “vulnerability” as the susceptibility to harm, which can be defined in terms of a population or location. In this context, actions to decrease vulnerability will decrease risk [18].

Impacts from drought and desertification can occur at the local or regional scales, but impacts can also be felt thousands of kilometers from the affected area. Land conflicts, for example could result in migration, and in turn this may overwhelm services (including health) in areas not prepared for the influx of migrants, leading to potential economic and political instability in these areas [12]. In arid or semiarid regions, where rainwater is scarce, an aggravation of a local situation may turn out to be invisible to local governments, limiting efficient decision-making. However, given the severity of its impacts, drought should be viewed as a priority environmental threat to human well-being [10].

## 2. Drought Impacts, the Case of Brazil

The International Disaster Database (EMDAT) is a repository of different types of disasters [1]. In order for an event to enter the EMDAT database, it has to follow one or more of the following criteria: 10 or more people reported killed; 100 people reported affected; a call for international assistance; or a declaration of a state of emergency [1]. In Brazil, for an event to enter the national disaster database, it has to follow the criteria of disruption of the functioning of the municipality or causing human, economic or environmental losses that exceed the ability of the affected municipality to cope using its own resources, thus requiring national assistance. Therefore, the number of events is much higher than what is reported in EMDAT. According to the Brazilian Atlas of Natural Disasters, between 1991 and 2010, there were close to 17,000 drought events recorded in 2944 municipalities in the country, making it the top disaster by type, with over 50% of total disaster events reported. Of a total of 96 million affected persons in these 20 years, 48 million (50%) were affected by drought (flash floods and other floods made up to 40%); and over a total of 2475 registered deaths, roughly 10% (257) were drought related. [19]. Two regions have been reporting drought events in the country. The southernmost portion of the country undergoes sporadic dry seasons leading to loss of crops and economic impacts, with a large number of affected persons but a small number of displaced and ill [19]. On the other hand, a large central portion of the northeast region comprises a permanently dry area, where extreme drought occurs periodically, affecting a larger population and causing population displacement and economic loses.

Brazil has defined an area in the Northeast as being semiarid. The inclusion criteria of semiarid are obtained when a municipality has an average annual pluviometric precipitation under 800 mm; or has a dryness index of under 0.5; or a drought risk greater than 60%. This area includes parts of nine (out of 27) States, and 1133 (out of 5565) municipalities, and a population of 22.6 million, which represents 12% of the Brazilian population [20]. The area roughly coincides with the biome known as the *Caatinga*. This is a fragile area, which is expected to change rapidly as a result of climate change. According to the Brazilian Panel on Climate Change [21], by 2040 the *Caatinga* biome should expect a temperature increase of 0.5–1.0 °C, and a precipitation decrease of 10%–20%. By 2100, temperature may increase up to 3.5–4.5 °C, with a precipitation decrease of up to 40%–50%. If this occurs, there is a risk that this part of Brazil will begin a process of desertification [21]. It is therefore of great importance for the health sector to understand this process, the problems it brings, and the actions needed to face it [22].

The population in the semiarid region has been living with and adapting to very adverse climatic conditions. However, this region presents an elevated level of vulnerability in front of additional pressures from climate change, which brings it to the limits of their adaptation capacity. As seasonal dry periods are expected, local population usually adopts water storage and agriculture practices to overcome water scarcity periods. If drought is severe or prolonged for more than two years, economic losses and health impact can be severely aggravated. The current environmental vulnerability is coupled with social and economic vulnerability, with a large proportion of the population living in poverty and extreme poverty. This complex problem is aggravated by a lack of infrastructure for water supply, which is limited to average water consumption of less than 20 liters per person, resulting in health vulnerability [23,24]. Adaptation is a key factor for coping with drought situations. Water supply is an example of climate sensitive decisions. During the wet seasons, households catch water from reservoirs, wells, or a home cistern. During prolonged droughts, alternative water sources are sought, sometimes far from the households, in potentially contaminated lakes and reservoirs. Institutional and community preparedness are critical to avoid making risk conditions worse [25].

**Figure 1:**
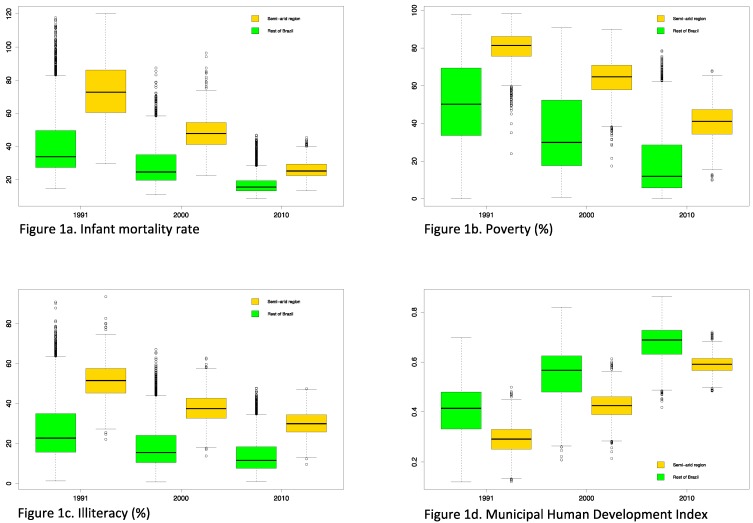
Trends in selected indicators for 5565 municipalities—in the semiarid region (1133) and the rest of Brazil (4432)—for 1991, 2000 and 2010: (**a**) infant mortality rate, (**b**) poverty rate, (**c**) illiteracy rate, and (**d**) Municipal Human Development Index.

**Figure 2:**
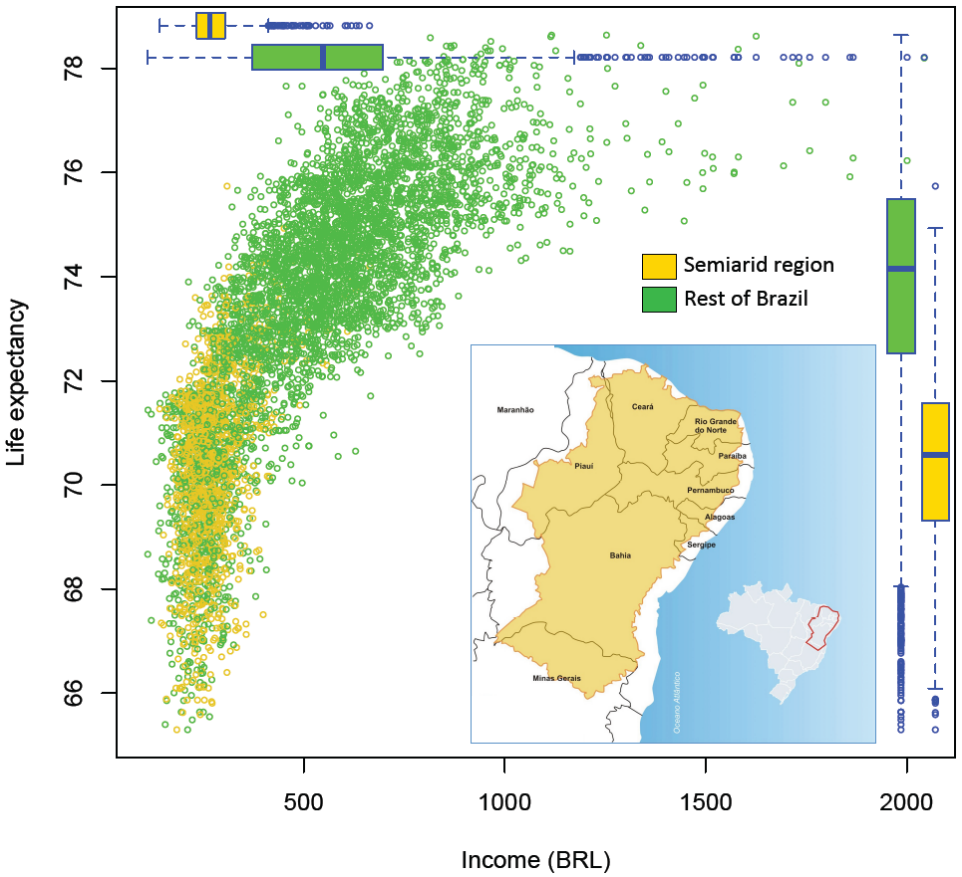
Life expectancy by average income, for 5565 municipalities—in the semiarid region (1133), and the rest of Brazil (4432)—for the year 2010, with boxplots showing the relative distributions (insert: Map of Brazil showing the area defined as Semiarid—Agencia Nacional de Aguas. http://memoria.ebc.com.br/agenciabrasil/sites/_agenciabrasil/files/gallery_assist/25/gallery_assist719504/ABr230413mapa%20Semirido.jpg. Creative Commons Atribuição 3.0 Brasil).

Population vulnerability in the semiarid region can be appreciated when comparing this region, with the rest of Brazil for key health and human wellbeing determinants. We obtained comparable census data for 1991, 2000 and 2010, for 5565 municipalities (identifying the 1133 municipalities corresponding to the semiarid region), and also an aggregated indicator, the municipal Human Development Index [26]. The database includes social, economic and environmental variables, aggregated at the municipal level. Aggregated data limits the analyses which can be performed but as the number of municipalities is large, it allows for interesting comparisons between regions, over time, and within regions (e.g., within the 1133 municipalities of the semiarid). Figure 1 shows time trends and differences for (a) infant mortality rate, (b) poverty rate (measured as the proportion of person living with less than BRL 140 per month, approximately USD 80 on 1 August 2010; exchange rate approximately at 1 USD = 1.75 Brazilian Real or BRL), (c) educational level (measured as the proportion of illiterate persons aged over 18 years) and (d) Municipal Human Development Index. Although there is a positive trend of improving wellbeing in all of Brazil, with important reductions in inequalities, the semiarid region appears worse off in terms of key indicators of health and wellbeing. Figure 2 shows life expectancy by income (average by municipality in BRL on 1 August 2010), for 2010. This figure shows important differences between the semiarid, with lower overall incomes and lower life expectancy as compared to municipalities in the rest of Brazil (boxplots).

This simple descriptive analysis shows a population with many characteristics of social vulnerability, living in a region with many characteristics of environmental vulnerabilities. As the environmental characteristics are expected to get worse with climate change [21], mitigation measures to address the health determinants and adaptation measures to tackle the current social determinants are urgent for this region. Climate and weather forecast can help local population to prevent economic losses and water shortage, for instance by informing what and when to cultivate for the next season. It also informs water managers to store water for forthcoming droughts [27], as these are expected due to their cyclical trend and their severity associated with the intensity of ENSO events [28,29]. There is also an opportunity to better integrate climate services for the benefit of public health [30]. Current climate models can predict severe droughts with some anticipation [31], allowing for planned health sector interventions.

## 3. Addressing the Health Impacts of Drought in Brazil

Given the slow onset and the large lag time to identify measurable health impacts, drought can be seen as a chronic emergency, which attracts less attention than an acute emergency, as is the case with floods. This has consequences in public health preparation and response [3]. Planning needs to be strengthened through the understanding of population vulnerability and insecure situations resulting from poverty, inappropriate soil and water management, a fragile local economy, subsistence mechanisms at risk, weak or ineffective governance for adaptation, institutional and population capacity, and the often limited resources available [8,13]. Faced with this challenge, the Ministry of Health in Brazil decided to establish a clear management process to implement actions of risk reduction, disaster management, and recovery and adaptation. This addresses a needed partnership between several areas including water resources, climate change, disaster risk reduction, social development, civil defense, and of course, health. These measures aim also at increasing resilience in order to face and recover from drought related risks [32,33]. As the health impact of droughts are mostly indirect and of long range, health surveillance systems must be reinforced, mainly during severe drought periods. Population displacement, water shortage and contamination, crop production failure, and cattle losses are intermediate events that must be monitored due to their potential health effects.

**Figure 3:**
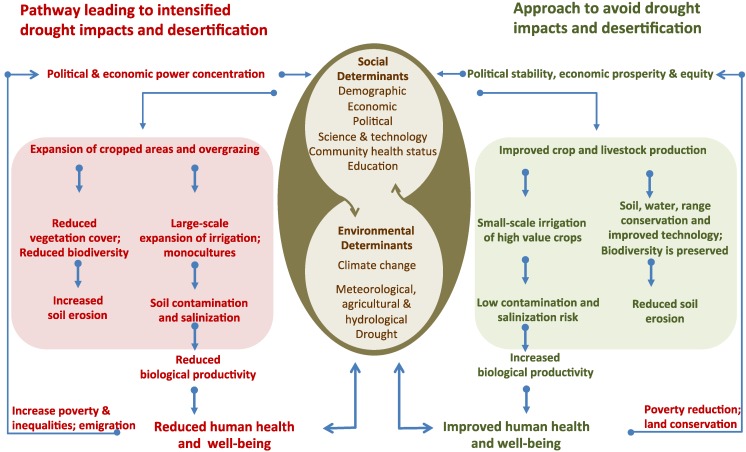
Drivers and pathways of drought and desertification, highlighting the central importance of social and environmental determinants of health and well-being. Adapted from [17,35].

Several factors have been identified as intervening in the development and severity of drought, and of their impacts on health and well-being as well as the environment and ecosystems. Regions, and within these, communities are affected differently by drought, and there are many intervening variables. Among these, and relevant to Brazil, we note the following [3,17,34]: (a) Socially determined—structure and capacity of existing water resources; socioeconomic development of the local communities; at risk population in the affected area; community vulnerability in front of social and environmental determinants; population health status; governance related to water use; population and local government resilience; environmental education programs; social programs and networks. (b) Environmentally determined—geophysical and environmental characteristics of the area; drought severity; water scarcity and contamination; soil contamination and salinization; land use change and degradation; loss of biodiversity; ecosystem degradation; inadequate crops; overgrazing; and the increasing impact of climate change. Figure 3 shows these factors within the process of intensification or control of drought impacts and desertification [17,35]. Table 1 provides a summary of relevant health conditions for the semiarid region in Brazil [3,17,34].

**Table 1:** Summary of relevant health conditions for the semiarid region in Brazil.

Systems and services	Human health
Access to drinking water (quality and quantity, unsafe water storage, limited water for hygiene)	Acute gastrointestinal diseases Water-borne and food-borne diseases Vector- and rodent-borne diseases, zoonoses
Food and nutrition (limited water for food hygiene, reduced or damaged crop yields, reduced health or death of animals and livestock)	Water-borne and food-borne diseases Malnutrition
Air quality (dust, drought related wild-fires)	Respiratory diseases (allergic rhinitis, asthma) Acute respiratory infections (bronchitis, sinusitis, pneumonia) Fungal infectious diseases (mycoses) Allergic reactions
Basic sanitation and hygiene (limited water for personal hygiene)	Infectious and parasitic diseases Skin infections
Mental health and behavior	Stress, anxiety, depression Behavioral changes, violence
Health services	Health service interruption Loss of medicines and personnel

**Figure 4:**
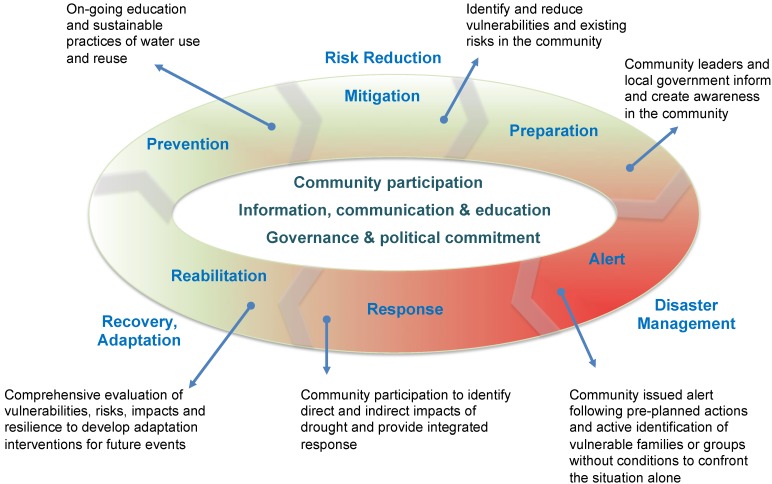
The role of the health sector in drought risk management. Framework with examples of community actions. Based on [13,33,36,37].

Disaster risk reduction in the health sector in Brazil follows a well-documented framework, which includes three stages: risk reduction, disaster management, and recovery. Within these, there are a series of actions, which go from prevention through mitigation, preparation, alert, response, rehabilitation and reconstruction [13,33,36,37]. This framework is adapted for drought management, and the steps are shown in Figure 4. What is key in this framework is the concept of adaptation. Given expected changes likely to make drought events more serious in the next few decades [21], it is necessary to address the development of adaptive capacity and resilience at the local level [38]. Local governance and complete community participation are necessary for successful and sustainable actions [39]. Table 2 provides a summary of proposed actions at each step adapted for the health impacts of droughts in Brazil (based on [13,33,36,37]). Note that given the special characteristic of drought, the last step, reconstruction, is not included as part of health sector actions (although it is recognized that in other type of events, health has a role in informing other sectors regarding its needs *vis-à-vis* reconstruction, e.g., of health facilities). In addition, important progress has been made in Brazil in reducing social and economic vulnerability to droughts. Although not highlighted specifically as health sector actions, programs for household water storage, expanding cisterns to collect rainwater before drought, building dams and drilling wells, financial support to agriculture, and ensuring a minimum income during drought are some examples of interventions with positive impacts on health and population well-being. Social programs such as the conditional cash transfer program known as *Bolsa Familia* and health programs such as Family Health have contributed to reduce the impact of the most recent drought (2011–2013), ensuring the country will never again experience catastrophic events such as the drought of 1877–1878 (500,000 deaths from drought and smallpox) or more recently the drought from 1979–1983 (tens of thousands deaths) [40,41].

**Table 2:** Drought risk management by the health sector in Brazil.

Risk reduction stage	
PREVENTION	On-going community and local government involvement, information and communication
	Promote educational measures and community actions for water conservation and measures to promote good nutrition
	Promote sustainable practices of water use and reuse
	On-going monitoring of water and food-borne diseases and selected non-communicable diseases in the communities at risk
	Follow-up the epidemiological profile of the community to identify adverse changes
	Promote capacity building of local health agents
MITIGATION	Work with local stakeholders to identify and reduce vulnerabilities and existing risks in the community
	Work with the local communities to develop measures aimed at minimizing risks and health impacts
	Promote health sector participation in public policy programs for water resource infrastructure
	Participate in inter-sectorial efforts to address drought impacts (e.g., with climate services to anticipate drought events)
PREPARATION	Assess the internal response capacity; identify local resources; and establish intra and inter-sectorial partnerships for action
	Participate in risk assessments, mapping, scenarios, to determine the severity of the problem from a health preparation perspective and to determine priority actions
	Implement the “Operative Committee of Health Emergencies*”, and establish the action plan.
	Work with community leaders and local government to inform and create awareness in the community
**Disaster management stage**	
ALERT	Issue alert following pre-planned actions and monitor its implementation
	Activate the “Operative Committee of Health Emergencies” and notify the event
	Actively identify families or groups without conditions to confront the situation alone
	Activate human and financial resources
RESPONSE	Provide for the health needs to the affected persons
	Intensify epidemiological, environmental and sanitary surveillance
	Monitor morbidity and mortality of direct and indirect impacts of drought
	Ongoing assessment of the response to determine future action
**Recovery and adaptation stage**	
REHABILITATION	Activate mechanisms to ensure the continuation of basic services, essential to the functioning of health facilities (e.g., water, energy)
	Activate specialized health care (e.g., for early identification and management of outbreaks)
	Activate psychosocial health care for the community and workers involved in the process
	Implement a comprehensive evaluation of vulnerabilities, risks, impacts and resilience to develop adaptation options for future events
* Operative Committee of Health Emergencies: A team formed by local stakeholders to organize and conduct risk management actions.	

## 4. Conclusions

Current social and environmental trends and expected future climate change impacts in semiarid regions present important challenges to the health sector. The health sector must ensure its active participation at all levels of government (Municipal, State and Federal), during inter-sectorial discussions on drought management. Although progress has been made in recent years, much more is needed to ensure health is seen as a key partner in drought risk management. Much can be achieved by ensuring a better collaboration between climate services and health services to strengthen risk management actions [30]. This would include investing in early warning systems for severe droughts based on climate models to inform the health sector, as well as other key sectors whose good performance also promotes good health (agriculture, water resource management, and disaster risk reduction).

The health sector also has a key role with regards to locally affected communities. There is need in promoting awareness of health risks and the social and environmental vulnerabilities of the different areas and communities, and to find mechanisms to increase the resilience of local communities and local government health services. Most importantly, the health sector must ensure that lessons learned from each event are implemented into adaptation plans. The health sector needs to ensure that all health risks, from the most immediate and visible (such as infant diarrheal diseases), through to the longer term yet visible impacts (such as malnutrition), to the less visible and delayed in time (such as mental health conditions), are fully included in its assessments and response.

Climate change, and the expected changes in the semiarid region in the next few decades [21], calls for a review of current programs, including health and the less researched issues such as gender differences, non-communicable diseases and mental health; and the planning of new interventions with local communities to ensure there will be sufficient and sustainable adaptive capacity and resilience, for a population already living with social inequalities and within the limits of environmental vulnerability.
